# Mobility Functional Status Ascertainment in Electronic Health Records using Large Language Models

**DOI:** 10.21203/rs.3.rs-7104310/v1

**Published:** 2025-07-29

**Authors:** Xingyi Liu, Muskan Garg, Heling Jia, Jennifer St. Sauver, Sandeep R. Pagali, Sunghwan Sohn

**Affiliations:** Mayo Clinic; Mayo Clinic; Mayo Clinic; Mayo Clinic; Mayo Clinic; Mayo Clinic

## Abstract

With global aging, assessing functional status is vital for precision medicine. Electronic Health Records (EHRs), particularly unstructured data, hold abundant information on patient mobility. This study explores using Large Language Models (LLMs) to extract and standardize mobility status from unstructured EHR data (i.e., clinical notes). We annotated 600 clinical notes from three health care institutions located in southeastern Minnesota and west-central Wisconsin, focusing on expressions of mobility and associated impairment. Leveraging the open-source Llama 3 model, we tested various prompting strategies—including zero-shot, few-shot, and task decomposition—and evaluated their performance. Error analysis showed that while the model sometimes inferred impairments without explicit evidence, most errors were clinically reasonable, often reflecting borderline or ambiguous cases. While considering reasonable inference as correct, at the patient-level, Mobility Extraction achieves a micro-average accuracy of 0.952 with an F1-score of 0.962, and Impairment Classification produces a micro-average accuracy of 0.912 and an F1-score of 0.948. A local, deterministic setup improved trustworthiness by ensuring consistent outputs, safeguarding privacy, and demonstrating cross-institution generalizability. These findings highlight the feasibility of LLM-based solutions for extracting mobility functional status from unstructured EHR data, supporting both clinical applications and research.

## 1 Introduction

The global demographic landscape is undergoing a shift, with a rapidly increasing proportion of older adults^1^. This demographic transition necessitates a fundamental change in healthcare, moving beyond a traditional focus on mortality to prioritize the maintenance of functional status and quality of life^2,3^. Functional status, broadly defined as an individual’s ability to perform activities necessary for daily living, is a powerful predictor of health outcomes, healthcare utilization, and overall well-being, particularly in older populations and those with chronic conditions^4–6^. Among the various domains of functional status, mobility – the ability to move oneself within an environment – is essential. Mobility limitations are strongly associated with increased risk of falls, hospitalization, institutionalization, reduced social participation, and diminished overall quality of life^7–9^. Accurate and timely assessment of mobility functional status is therefore essential for effective geriatric care, rehabilitation, and the development of personalized interventions.

Traditionally, mobility functional status has been assessed through standardized questionnaires (e.g., self-reported activity limitations) and performance-based measures (e.g., Timed Up & Go test, gait speed assessment)^10^. However, a rich source of information on patient mobility also resides within electronic health records (EHRs)^11,12^. Clinicians routinely document observations, patient reports, and assessments related to mobility in clinical notes. Unfortunately, this information is typically recorded in unstructured or semi-structured text, making it difficult to extract and analyze systematically. The complexity, variability, and nuanced language used in clinical documentation pose a significant challenge to traditional data extraction techniques. This represents a major barrier to leveraging the wealth of information contained within unstructured EHRs to improve the understanding and management of mobility limitations. Furthermore, manual chart review, the traditional method for extracting information from unstructured text, is prohibitively time-consuming and expensive for large-scale research or routine clinical use^13^.

This study proposes a novel approach to address this challenge by employing Large Language Models (LLMs) to automatically ascertain and standardize mobility functional status from unstructured EHR data (i.e., clinical notes). LLMs, with their advanced capabilities in natural language understanding and generation, offer a powerful means of processing and interpreting complex textual data^14,15^. Prior research has demonstrated the potential of natural language processing (NLP) techniques, including earlier machine learning approaches and rule-based systems, for extracting information related to functional status and mobility from clinical text^16,17^. For example, Bales et al. modified an existing NLP system to automate the assignment of selected International Classification of Functioning, Disability, and Health (ICF) codes^18^ by updating the lexicon and coding tables^19^. Agaronnik et al. employed the ClinicalRegex NLP software to search EHRs for functional status documentation, utilizing an ontology with five keyword categories to identify pertinent information^20^. Newman-Griffis et al. applied NLP techniques to analyze patient functioning information in clinical documents related to federal disability benefit claims from the U.S. Social Security Administration, achieving robust performance with an F1-score exceeding 80% on two datasets^21^. More recently, Fu et al. introduced FedFSA, a hybrid and federated NLP framework designed to extract functional status information from EHRs across multiple healthcare institutions^22^. These earlier efforts, while showing promise, often faced limitations in terms of generalizability, scalability, and the ability to handle the full complexity and ambiguity of clinical language. Recent advances in LLM architecture and training have demonstrated remarkable performance on a wide range of NLP tasks, including information extraction, question answering, and text summarization, overcoming many of the limitations of prior NLP approaches^23–25^. These capabilities make LLMs particularly well-suited to the task of extracting structured information from unstructured clinical text, where contextual understanding and the ability to handle ambiguity are crucial. By leveraging the power of an open-source LLM, Llama 3^26^, we aim to develop a robust and scalable pipeline that can automatically extract, classify, and standardize mobility-related information from unstructured clinical text.

The contribution of this study is fourfold. First, it develops a comprehensive annotation scheme grounded in the ICF framework to identify and classify expressions related to mobility functional status in clinical notes. Second, it evaluates the performance of Llama 3 in extracting and classifying mobility impairment status by employing various prompt engineering techniques, such as zero-shot learning, few-shot learning, error-informed prompt refinement, and task decomposition. Third, it assesses the generalizability of the LLM-based approach across different healthcare institutions. Finally, it identifies common error patterns and evaluates the overall trustworthiness of the model.

We hypothesize that LLMs with well-constructed prompt engineering strategies can effectively extract mobility functional status information from unstructured EHR data, providing a scalable solution for both clinical practice and research. The successful implementation of this approach has the potential to significantly enhance precision medicine by enabling more timely and personalized interventions, improving the monitoring of patient progress, and facilitating large-scale research on mobility and aging. This, in turn, could lead to improved clinical decision-making, better resource allocation, and ultimately, improved quality of life for older adults and individuals with mobility limitations.

The remainder of this paper is organized as follows. Section 2 presents the results of our experiments, including performance metrics and error analysis. Section 3 discusses the implications of our findings, limitations of the study, and directions for future research. Section 4 details the materials and methods used in this study, including the study population, annotation process, LLM selection, and prompt engineering strategies.

## 2 Results

### 2.1 Data analysis

**Figure 1:**
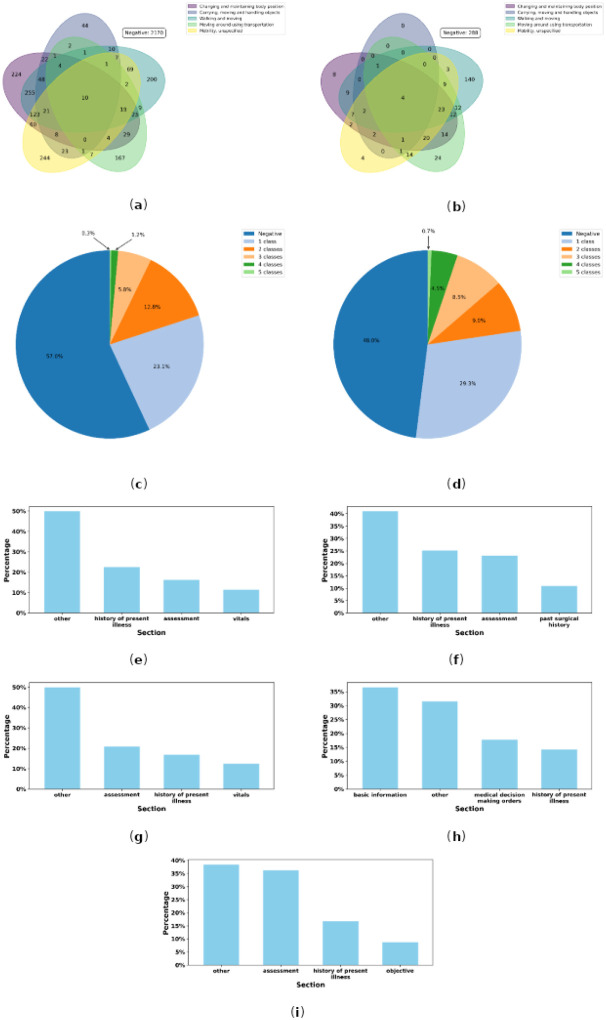
Analysis of Mobility Classes at Section and Note Levels and Distributions of Sections for Mobility Classes. **(a)** Section-level Venn diagram showing the number of clinical note sections (n = 3,810) containing each combination of the five mobility functional status classes (Negative = no classes mentioned). **(b)** Note-level Venn diagram showing the number of clinical notes (n = 600) containing each combination of the five classes. **(c)**Pie chart of the proportion of sections mentioning 0–5 classes: 57.0% none, 23.1% one, 12.8% two, 5.8% three, 1.2% four, 0.3% five. **(d)** Pie chart of the proportion of notes mentioning 0–5 classes: 48.0% none, 29.3% one, 9.0% two, 8.5% three, 4.5% four, 0.7% five. **(e-i)** Distribution of sections for “Changing and maintaining body position”, “Carrying, moving, and handling objects”, “Walking and moving”, “Moving around using transportation” and “Mobility, unspecified”, respectively, showing the three most frequent sections.

**Figure 2:**
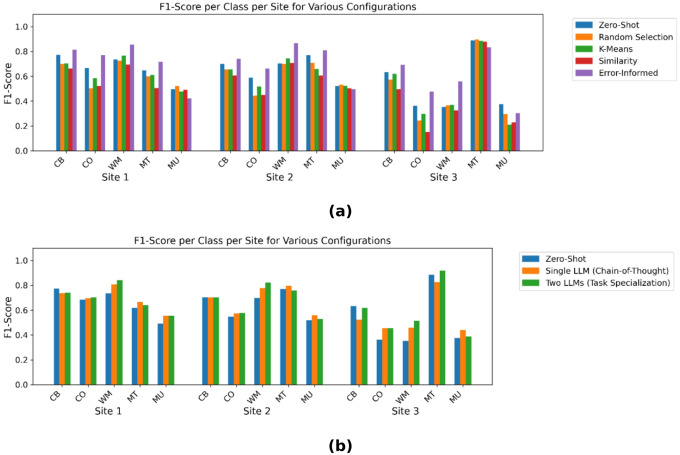
Section-level F1-scores for mobility extraction across five functional status classes and three institutions under different prompt and task configurations. **(a)** Comparison of zero-shot baseline, three few-shot learning strategies and error-informed prompt refinement approach. **(b)** Comparison of zero-shot baseline and two task decomposition setups. (CB: Changing and maintaining body position, CO: Carrying, moving, and handling objects, WM: Walking and moving, MT: Moving around using transportation, MU: Mobility, unspecified).

Figure 1 provides a comprehensive analysis of mobility classes at both the section and note levels. In Fig. 1(a), the number of sections corresponding to each combination of the five mobility classes is shown. It reveals that 10 sections mention all five classes, whereas 2,170 sections mention none. Figure 2(b) extends this analysis to the note level, illustrating the number of notes corresponding to each combination of the five mobility classes. It indicates that four notes mention all five classes, while 288 notes do not mention any.

Additionally, Fig. 1(c) presents a pie chart showing how many mobility classes each section contains. As shown, 57% of sections mention no mobility classes, 23.1% mention one, 12.8% mention two, 5.8% mention three, 1.2% mention four, and 0.3% mention all five classes. Similarly, Fig. 1(d) illustrates how many mobility classes appear in entire notes. It reveals that 48% of notes do not mention any mobility classes, 29.3% mention one, 9.0% mention two, 8.5% mention three, 4.5% mention four, and 0.7% reference all five.

A combined overview was created to show how often each mobility class was mentioned, along with the distribution of impairment statuses (Impaired vs. Unimpaired). At the section level, the most frequently mentioned mobility class is “Changing and maintaining body position”, appearing in 853 sections; of these, 608 indicate Impaired status, while the remainder suggest Unimpaired. In contrast, the least frequently mentioned mobility class at the section level is “Carrying, moving and handling objects”, noted in only 203 sections, 156 of which suggest Impaired status. At the note level, the most frequently mentioned mobility class is “Walking and moving”, found in 222 notes; 197 of these suggest Impaired status, with the remainder indicating Unimpaired. Meanwhile, the least frequently mentioned mobility class at the note level is “Carrying, moving and handling objects”, referenced by only 11 notes, 8 of which suggest Impaired status.

Figure 1 (e)–(i) illustrates the distribution of sections for each of the five mobility classes. For each class, we identified the three most frequently mentioned sections and grouped all remaining sections under “other”. The results showed that “history of present illness” ranks among the top three for all five mobility classes, while “assessment” appears in the top three for four of the five classes, with the exception of “Moving around using transportation”.

### 2.2 Baseline Performance with Zero-Shot Learning

Table 1 presents the baseline section-level performance on Task 1 (Mobility Extraction) and Task 2 (Impairment Classification). This evaluation was conducted across the three participating institutions and five mobility functional status classes. Task 1 metrics include class- and institution-specific precision, recall, and F1-scores. The micro-average F1-scores across all mobility classes are 0.663, 0.638, and 0.516 for site 1, site 2, and site 3, respectively. Overall, these baseline metrics varied by class and location, with some classes performing consistently well while others presented room for improvement. They serve as a reference point against which more complex prompting strategies and configurations can be compared.

Regarding Task 2 (Impairment Classification), the class “Walking and moving” demonstrated best performance with F1-scores ranging from 0.930 to 0.964 across three sites. The class “Moving around using transportation” achieved the lowest performance with F1-scores ranging from 0.649 to 0.755 across sites. The micro-average F1-scores across all mobility classes are 0.888, 0.857, and 0.833 for sites 1–3 respectively.

### 2.3 Comparison of Few-Shot Learning and Error-Informed Prompt Refinement Approaches

Figure 2(a) presents a comparison of section-level F1-scores for Mobility Extraction across five mobility functional status classes and three institutions under five configurations: the baseline zero-shot learning method, three few-shot learning methods (Random Selection, K-Means Error Clustering Selection, and Similarity-Based Selection), and the Error-Informed Prompt Refinement strategy. In each bar cluster, the bars represent the section-level F1-scores for a specific class and institution combination.

Overall, while most few-shot configurations underperform relative to the zero-shot baseline, the extent of this performance gap varies by both class and institution. In some cases, few-shot learning slightly outperforms the baseline, whereas in others the decline is more pronounced. One possible explanation is that longer prompts can lead the model to “forget” crucial information provided earlier in the context. In few-shot settings, the additional examples may dilute or overshadow key information like definitions of mobility classes from the beginning of the prompt.

Notably, the Error-Informed Prompt Refinement approach yields significant improvements in most scenarios, with the exceptions of the “Mobility, Unspecific” class at all the three sites and the “Moving around using transportation” class at Site 3. These per-class, per-institution comparisons provide valuable insights into the conditions under which each method is most effective.

### 2.4 Task Decomposition Method Performance and Integration

Figure 2(b) illustrates the comparison of the zero-shot baseline F1-scores for Mobility Extraction against those obtained using the two task decomposition methods at section level: (1) Single LLM with Two-Task Prompt (Chain-of-Thought Prompting) and (2) Two LLMs with Task Specialization.

As shown in Fig. 2(b), neither task decomposition approach consistently outperforms the zero-shot baseline across all classes and institutions—sometimes exceeding the baseline, and other times falling short. We then integrated the two task decomposition methods with the top-performing Error-Informed Prompt Refinement strategy. Upon re-evaluation, this combined approach did not consistently surpass the performance of the Error-Informed Prompt Refinement strategy applied alone across all classes and institutions. These findings suggest that more complex configurations do not necessarily lead to better outcomes. Overall, our best configuration remains the standalone Error-Informed Prompt Refinement strategy.

### 2.5 Best Performance with Error-Informed Prompt Refinement

The section-level performance of the Error-Informed Prompt Refinement is summarized in Supplementary Table S2. The micro-average F1-scores across all mobility classes are 0.729, 0.723, and 0.608 for sites 1, 2, and 3, respectively.

Regarding Task 2 (Impairment Classification) with the Error-Informed Prompt Refinement, notably, the “Walking and moving” class performed best, with F1-scores between 0.913 and 0.957 across the three sites. The micro-average F1-scores across all mobility classes are 0.832, 0.814, and 0.785 for sites 1, 2, and 3, respectively.

Supplementary Table S3 presents a comprehensive summary of the section-level, note-level and patient-level performance with the Error-Informed Prompt Refinement configuration for both tasks, aggregated across all institutions. The micro-average F1-scores for Task 1 and Task 2 were 0.695 and 0.815 at the section-level, 0.819 and 0.849 at the note-level and 0.876 and 0.897 at the patient-level, respectively.

### 2.6 Error Analyses

This section presents major error themes identified during our analyses. We highlight key areas for refinement and inform future enhancements to model performance and reliability. Major error themes include missing functional status context, lack of medical knowledge, ambiguous definition, issues of certainty, exclusion and inference.

- Inference: Inference errors are a common source of false positives. For example, when a section mentions “right lower leg pain that began approximately 3 years ago with activities”, the LLM inferred an impairment in “Changing and maintaining body position”. Although clinically reasonable, such inferences were excluded during annotation because there was no direct mention of a limitation in that mobility class. Similarly, phrases like “right hand pain, hand swelling” led the model to infer an impairment in “Carrying, moving and handling objects”, and “has more knee pain with the exercises” prompted an inference of impairment in “Walking and moving”. In these cases, the absence of explicit evidence for a functional limitation necessitated their exclusion from impaired status assignments.

Another error pattern arises from the handling of non-specific symptoms. When sections mention term such as “dizziness”, “headaches”, “vertigo”, “weakness”, or “debility”, the LLM often assigned an impairment in the “Mobility, unspecified” class. However, in the absence of a direct reference to a functional limitation (e.g., difficulty ambulating or transferring), our annotation guidelines mandate that such general symptoms be excluded from mobility impairment classification. This overgeneralization by the LLM contributes to false positives and decreases the precision for the “Mobility, unspecified” class.

- Ambiguous Definitions: Some mobility concepts share similar semantics, leading to overlapping or inconsistent classifications. For instance, repeated falls were frequently classified by the LLM as impairments in “Walking and moving”. However, our clinical rationale dictated that such cases should be classified under “Changing and maintaining body position” to better capture issues with postural control. Similarly, mentions of wheelchair use were often split between “Walking and moving” and “Moving around using transportation”. To enhance clarity and consistency, we consolidated wheelchair-related instances exclusively under “Moving around using transportation”.
- Missing Definition: The class “Mobility, unspecified” remained problematic across tasks. This challenge arises primarily from the lack of a precise ICF definition for this class, rendering its scope inherently ambiguous. In practice, this class is meant to capture general aspects of mobility—such as everyday activities and exercise—but without clear boundaries. For example, when a clinical note states, “patient returned fully to yoga,” it implies an impairment within this class.
- Exclusion: Documentation pertaining to treatment plans, discussions, suggestions, instructions, recommendations, goals or advice is generally not considered indicative of a patient’s mobility functional status. This rule applies unless such documentation explicitly mentions that the patient is expected to improve in a specific mobility class, which in turn implies that an impairment exists. For example, if a note states that ‘the patient will improve in walking ability with physical therapy’, this suggests an underlying impairment in that area and should be annotated accordingly. Otherwise, routine mentions of treatment-related content are excluded from contributing to the functional status classification. However, we observed that the LLM sometimes struggles to exclude such content even when the prompt explicitly directs it to do so.
- Limitations in Medical Knowledge: LLMs trained on general corpora may lack the nuanced medical expertise required for certain assessments. For instance, the Focus on Therapeutic Outcomes (FOTO) system is a specialized web-based assessment where a low final score (ranging from 0 to 100) indicates impairment in the “Mobility, unspecified” class. Similarly, the Patient Specific Functional Scale (PSFS) is widely used to capture patient-specific reports of functional ability. In clinical practice, a low current PSFS score—or an expectation of future improvement—serves as a direct indicator of impaired functional status in the “Mobility, unspecified” class. The Timed Up and Go (TUG) Test is a standardized assessment for evaluating mobility and the risk of falls. However, our analysis revealed that the LLM does not consistently integrate these quantitative assessments into its interpretation of clinical notes. As a result, numerical scores or standardized test results might be inferred indirectly.

As noted in inference error analysis, some false positive inference cases are clinically reasonable to be considered correct (i.e., true positive). Therefore, we also evaluated the performance of the LLM after treating such reasonable inferences as correct. Table 2 presents a comprehensive summary for both task 1 and task 2, aggregated across all institutions under this assumption. The performance was improved compared to the original results (Supplementary Table S3); the micro-average F1-score for task 1 and task 2 at the section-level increased to 0.890 and 0.878, respectively (originally 0.695 and 0.815), 0.942 and 0.925 at note-level, 0.962 and 0.948 at patient-level.

### 2.7 Trustworthiness Analyses

LLMs used in clinical contexts must demonstrate trustworthiness across several dimensions, including reliability, safety, and robustness. In this section, we evaluate our approach and identify how our methods and configurations contribute to these attributes.

- Reliability: We configured the LLM with a temperature setting of 0, ensuring deterministic responses. By eliminating randomness in the generation process, the model’s outputs are reproducible: repeated queries yield consistent results. Additionally, our error analysis revealed that although the LLM does make mistakes, these errors are generally “reasonable” rather than erratic or nonsensical. For instance, the model might misclassify a borderline case of mobility impairment, but it does not invent implausible conditions. Such understandable, bounded errors indicate that the model’s performance is stable and predictable, which is critical for building clinical trust.
- Safety: Maintaining patient privacy and data confidentiality is paramount in healthcare applications. To this end, we deployed the LLM on secure local servers, preventing any transfer of protected health information (PHI) to external environments. By avoiding reliance on remote or third-party cloud services, we mitigated the risk of unintended data exposure and ensure compliance with privacy regulations. This closed-loop infrastructure provides a safer environment for processing sensitive clinical notes.
- Generalizability: A trustworthy LLM must demonstrate robust performance across various clinical contexts. Although the training dataset was sourced from a single site, our analysis shows that refinements—such as introducing examples or error informed instructions—improved the model’s performance not only at the original site but also at two additional independent sites. This form of transfer learning highlights the model’s adaptability and generalizability, suggesting that improvements identified in one environment can successfully translate to others. Consequently, the LLM remains effective under slightly shifted data distributions, thereby increasing its overall trustworthiness as a tool for broad clinical application.

By addressing each of these dimensions—ensuring reproducibility and logical consistency, protecting patient data privacy, and demonstrating cross-institutional adaptability—we underscore the trustworthiness of our LLM-based annotation pipeline.

## 3 Discussion

This study demonstrates the feasibility of employing LLMs with well-constructed prompt engineering to automatically extract and standardize mobility functional status information from unstructured EHR data. By leveraging an open-source LLM (Llama 3) alongside tailored prompt engineering strategies—including zero-shot, few-shot, and error-informed prompt refinement—we developed a pipeline that reliably identifies and classifies mobility-related expressions across diverse clinical note sections.

Our evaluations across three healthcare institutions reveal that, while performance varies among different mobility classes, the refined prompt strategy significantly enhances overall accuracy and consistency. The Error-Informed Prompt Refinement configuration was designed to address specific shortcomings observed in the baseline model by incorporating feedback from previous errors. By analyzing misclassifications and instances of low precision, the model was able to adapt its prompts to better clarify ambiguous cases. This adjustment was particularly beneficial for classes that initially suffered from high variability, such as “Carrying, moving, and handling objects” and “Walking and moving” in the Mobility Extraction task. For Impairment Classification, while the top-performing class (“Walking and moving”) maintained high performance, the refinement process contributed to a more consistent performance for classes like “Changing and maintaining body position”. The narrower range of F1-scores (0.793–0.851) in this setting suggests that error-informed adjustments helped reduce performance fluctuations across institutions.

The Error-Informed method sometimes exhibited a trade-off where enhancements in precision for some classes came at the expense of recall. For example, in Impairment Classification, although the precision improved for class “Mobility, unspecified”, the corresponding recall dropped, which in turn affected the F1-score. This indicates that while the refined prompts can be more targeted, they may sometimes become overly conservative, missing some correct instances.

Portability of the model across different healthcare institutions remains an important area for further exploration. Our development took place at an academic research center (Site 1) and was tested at two other institutions (Sites 2 and 3) that include data from community-based real-world practices to validate generalizability. In future work, we aim to test different combinations of development and validation, and also expand data sources with more diverse patient populations, clinical note structures, and documentation styles. This would allow us to systematically assess model robustness and understand the impact of data heterogeneity on performance metrics.

Another limitation of our approach is that we did not fine-tune the LLM on the annotated dataset. Although fine-tuning has the potential to improve performance, our annotated dataset was relatively small, and fine-tuning large models typically requires substantial amounts of labeled data to avoid overfitting. In addition, fine-tuning can be computationally expensive and may increase the risk of overfitting to specific institution documentation styles. Instead, we relied on prompt-engineering strategies that can be more data-efficient and flexible. However, future research could explore lightweight fine-tuning methods (e.g., LoRA or adapter-based approaches) if larger annotated corpora become available.

Moreover, the deterministic configuration and secure local deployment of the model address key concerns regarding reproducibility and patient data privacy, further reinforcing the pipeline’s trustworthiness. Despite challenges—such as handling ambiguous language and balancing precision with recall—the findings indicate that LLMs can serve as scalable and generalizable tools for clinical applications. Future work may further explore additional domains of trustworthiness, such as fairness and interpretability, to provide an even more comprehensive assessment of the model’s reliability and suitability for clinical environments.

Longitudinal validations in real-world settings will be essential for fully integrating these methods into clinical decision-making processes, ultimately contributing to improved patient care and personalized intervention strategies. Overall, our work lays a promising foundation for advancing the automated extraction of functional status data, highlighting the transformative role of LLMs in modern healthcare analytics.

## 4 Methods

### 4.1 Data Preparation

This study included three institutions an academic referral medical center and two community-based practices in the upper midwest. Agreements between these institutions preclude direct comparisons of data; therefore, the sites are designated by number (sites 1–3). The annotation guidelines were developed using definitions from ICF to annotate mobility functional status data in clinical notes. We considered five mobility functional status classes, each comprising one or more ICF subclasses. Supplementary Table S1 lists these classes alongside their corresponding ICF codes and subclasses.

We restricted our analysis to five primary mobility classes, rather than using their more granular ICF subclasses. This decision was driven by two practical considerations. First, our annotation process revealed a high degree of semantic overlap among the ICF subclasses, making it difficult to obtain reliable annotation for those narrower categories. As a result, we consolidated the ICF-based taxonomy into five major classes as defined in ICF to enhance labeling consistency and reduce confusion during both manual annotation and automated extraction. Second, LLMs may struggle with overly fine-grained distinctions in clinical text, especially if subclass definitions overlap or are ambiguously documented.

To facilitate focused and coherent annotation, each clinic note was divided into sections. Clinical notes at Site 1 include a variety of sections, such as History of Present Illness, Past Medical/Surgical History, Physical Examination, Diagnosis and more. We chose section-level analysis for several reasons. First, clinical notes can be lengthy, and feeding entire notes into a LLM risks exceeding context length limits—potentially truncating important information. Second, sections typically focus on specific aspects of a patient’s health (e.g., History of Present Illness, Assessment), which makes them natural, semantically coherent units for targeted extraction tasks. Third, section-level segmentation facilitates more precise few-shot prompts and helps the model focus on local contextual cues relevant to mobility. Finally, working at the section level can improve interpretability, since clinicians often review notes by scanning sections, allowing direct alignment of the LLM’s output with established clinical documentation practices.

Within each section, a trained annotator manually identified and labeled mobility classes with related expressions and assigned the appropriate impairment statuses. The annotator assigned “Impaired” if the section describes difficulties, challenges, limitations, potential issues, or impairments, and “Unimpaired” if the section explicitly describes normal function or abilities. If a section did not receive either “Impaired” or “Unimpaired,” this indicated that there was no mobility-related information present in that section. Additional details on the annotation process and guidelines can be found in the Supplementary Section.

In total, 600 notes were annotated. Of these, 200 were physical therapy (PT) or occupational therapy (OT) notes from Site 1, 200 were PT/OT notes from Site 2, and 200 were unrestricted clinic notes from Site 3. PT/OT notes typically provide abundant information regarding mobility, while including general clinic notes from Site 3 helps demonstrate the method’s broader applicability. After splitting the notes into sections, the dataset comprised 3,810 sections (1,153 in Site 1, 1,075 in Site 2, and 1,582 in Site 3). On average, each section contained 826 characters, with section lengths averaging 932 characters in Site 1, 847 in Site 2, and 7,334 in Site 3. A single section may contain information relevant to multiple mobility classes or none.

### 4.2 Experiment

For prompt development, 200 sections were randomly selected from Site 1 to form a training dataset; the remaining sections served as the test set. Performance was evaluated using precision, recall, and F1-score metrics, both class-specific and institution-specific, across various experimental configurations.

**Figure 3:**
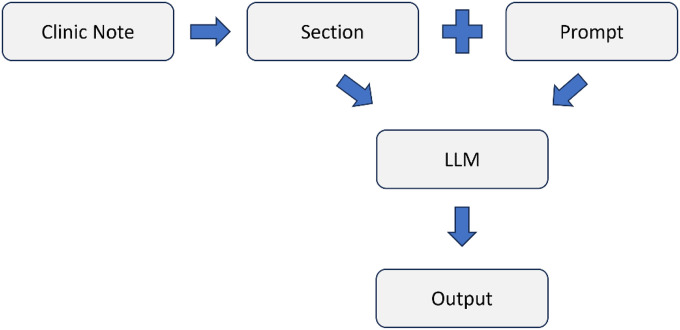
LLM-based Annotation Pipeline. Clinical notes are segmented into discrete sections. Each section is embedded in a task-specific prompt defining the five mobility classes. The LLM processes the prompt and outputs.

An LLM-based annotation pipeline was developed to extract mobility functional status information. The pipeline consists of three core components: (1) the LLM, (2) a task-specific prompt combined with a clinical note section, and (3) a post-processing step that converts the LLM’s text output into structured data. As shown in Fig. 3, the process begins with dividing the clinical notes into distinct sections. Each section is then embedded within a task-specific prompt explicitly designed for mobility functional status extraction. The LLM processes the prompt and generates a textual response based on its analysis and interpretation, and finally, a post-processing step transforms the LLM’s generative text output into structured objects. Specifically, for each section, the LLM produces, for each of the five mobility classes, a single label—either “Impaired” if there is evidence of limitation, “Unimpaired” if normal function is described, or “None” if the section contains no information about that class.

Llama 3, an advanced open-source LLM developed by Meta AI, was employed for this task. Llama 3 provides enhanced efficiency and performance. Integrating Llama 3 into a local server environment ensured both patient data privacy and the use of powerful computational resources for annotation tasks. This setup allowed for the handling of sensitive medical information within a secure infrastructure. The open-source nature of Llama 3 further facilitated customization and adaptability to specific project requirements.

#### 4.2.1 Performance Evaluation

We calculate performance at the class level. For each mobility class, we first evaluate Mobility Extraction by considering any section labeled “Impaired” or “Unimpaired” for that class as “Mentioned” and all other sections as “Not Mentioned.” Treating “Mentioned” as the positive class and “Not Mentioned” as the negative class, we compute precision, recall, and F1-score by comparing the LLM’s predicted Mentioned/Not Mentioned labels against the human annotations for that class. Next, for Impairment Classification, we restrict our analysis to those sections confirmed as “Mentioned.” Within this subset, the LLM’s output—distinguishing between “Impaired” and “Unimpaired”—is compared to the human reference label. In other words, once a section is known to address the mobility class, we assess whether the LLM’s choice of “Impaired” versus “Unimpaired” matches the gold-standard annotation, and we calculate an F1 - score for each class with “Impaired” as the positive label.

### 4.3 Prompting Engineering

#### 4.3.1 Zero Shot Learning

In the zero-shot configuration, the prompt includes only the core task description, the definitions of all relevant mobility classes, a general instruction, and a final question. It does not provide any examples. Under these conditions, the model relies solely on the given prompt, without guidance from sample inputs or outputs. Evaluating zero-shot performance establishes a baseline against which subsequent methods can be compared.

#### 4.3.2 Few Shot Learning

Few-shot learning leverages a small number of example cases embedded in the prompt to improve the model’s reasoning and output quality. Unlike zero-shot prompting, few-shot prompts present the model with illustrative examples—each accompanied by detailed explanations—to better convey the task’s underlying reasoning steps.

In this study, a five-shot configuration was used, with the selected examples spanning a variety of scenarios to promote more nuanced understanding. Three strategies were tested for choosing these examples from the training dataset:

- Random Selection: Five samples are chosen at random.
- K-Means Error Clustering Selection: We first identified the errors that occurred under zero-shot conditions on the training dataset. We then applied the k-means clustering algorithm to group these error cases into five clusters. From the center of each cluster, we selected one representative sample, thereby focusing on common mistakes.
- Similarity-Based Selection: For each test case, we identified the top five most similar training samples using a K-nearest neighbors approach. These closely related examples serve as relevant reference points that closely match the characteristics of the test input.

Through these strategies, we aimed to discover which method of choosing examples leads to the greatest performance improvement, demonstrating how few-shot learning can be optimized to achieve more accurate and reliable results.

#### 4.3.3 Error-Informed Prompt Refinement

A systematic error analysis of the zero-shot results was conducted to identify patterns in the model’s mistakes, such as common misclassifications or omissions. We manually examined all false positive and false negative outputs across sections in the training set, catalogued the most common sources of error, and distilled these into a set of explicit “exclusion” and “inclusion” rules. These rules were then injected as additional instructions into the prompt so that the LLM would be guided toward avoiding the same mistakes. For example, whenever the section mentioned “patient will begin home exercise program”, the model tended to treat these as evidence of impairment—even though they are plans or instructions. To address this, we added explicit instructions telling the model to ignore any text that describes a treatment plan, recommendation, or exercise regimen.

#### 4.3.4 Task Decomposition

Our overarching goal was to extract expressions related to a patient’s mobility functional status and classify their impairment status (impaired vs. non-impaired). To potentially improve performance, we divided the task into two subtasks: Mobility Extraction and Impairment Classification. This decomposition enables a clearer, more focused approach and allows for specialized handling of each step. To implement this, we explored two different setups:

- Single LLM with Two-Task Prompt (Chain-of-Thought Prompting): A single LLM is guided sequentially through the two subtasks with a multi-step prompt. The prompt first instructs the model to assess whether the section contains mobility-related descriptions for each class, then directs it to classify the impairment status. By leveraging a chain-of-thought process, this setup encourages methodical, stepwise reasoning.
- Two LLMs with Task Specialization: Alternatively, this approach employs two dedicated LLMs, each focusing on a single subtask. The first LLM determines whether relevant mobility descriptions are present. The second LLM then uses those determinations to classify impairment status. By dividing responsibilities in this manner, each model can specialize its role.

## Figures and Tables

**Table 1:** Performance of Task 1 (Mobility Extraction) and Task 2 (Impairment Classification) Across Mobility Classes and Institutions with Zero-Shot Learning at Section Level.

Class	Site 1				Site 2				Site 3			
	Task 1			Task 2	Task 1			Task 2	Task 1			Task 2
	P	R	F1	F1	P	R	F1	F1	P	R	F1	F1
Changing and maintaining body position	0.745	0.802	0.772	0.837	0.627	0.789	0.699	0.805	0.605	0.665	0.634	0.757
Carrying, moving, and handling objects	0.589	0.768	0.667	0.894	0.577	0.602	0.589	0.814	0.273	0.545	0.364	0.857
Walking and moving	0.584	0.989	0.734	0.964	0.542	0.997	0.703	0.930	0.214	1.000	0.352	0.962
Moving around using transportation	0.688	0.611	0.647	0.649	0.771	0.771	0.771	0.718	0.931	0.849	0.888	0.755
Mobility, unspecified	0.341	0.905	0.495	0.872	0.357	0.960	0.521	0.836	0.252	0.730	0.375	0.914
Average	0.534	0.874	0.663	0.888	0.500	0.880	0.638	0.857	0.381	0.798	0.516	0.833

* P: Precision, R: Recall, F1: F1-score.

**Table 2:** F1-score of Two Tasks Across Mobility Classes Aggregated Across All Institutions with Error-Informed Prompt Refinement When Considering Reasonable Inference as Correct.

Class	Section-Level		Note-Level		Patient-Level	
	Task 1	Task2	Task 1	Task2	Task 1	Task 2
Changing and maintaining body position	0.867	0.866	0.930	0.929	0.956	0.951
Carrying, moving, and handling objects	0.877	0.878	0.909	0.902	0.907	0.917
Walking and moving	0.948	0.951	0.982	0.984	0.989	0.982
Moving around using transportation	0.877	0.730	0.939	0.750	0.966	0.824
Mobility, unspecified	0.833	0.810	0.916	0.903	0.963	0.966
Average	0.890	0.878	0.942	0.925	0.962	0.948

* F1: F1-score.

## Data Availability

The data used for this work was from electronic health records which include identifiable data and thus cannot be shared due to privacy and legal reasons.
